# A geometric scaling model for assessing the impact of aneurysm size ratio on hemodynamic characteristics

**DOI:** 10.1186/1475-925X-13-17

**Published:** 2014-02-17

**Authors:** Yunling Long, Hongyu Yu, Zhizheng Zhuo, Ying Zhang, Yang Wang, Xinjian Yang, Haiyun Li

**Affiliations:** 1 School of Biomedical Engineering, Capital Medical University, Beijing 100069, China; 2 Beijing Neurosurgical Institute, Beijing Tiantan Hospital, Beijing 100050, China

**Keywords:** Intracranial aneurysm, Scaled models, Aneurysm Size Ratio, Hemodynamics

## Abstract

**Background:**

The intracranial aneurysm (IA) size has been proved to have impacts on the hemodynamics and can be applied for the prediction of IA rupture risk. Although the relationship between aspect ratio and hemodynamic parameters was investigated using real patients and virtual models, few studies focused on longitudinal experiments of IAs based on patient-specific aneurysm models. We attempted to do longitudinal simulation experiments of IAs by developing a series of scaled models.

**Methods:**

In this work, a novel scaling approach was proposed to create IA models with different aneurysm size ratios (ASRs) defined as IA height divided by average neck diameter from a patient-specific aneurysm model and the relationship between the ASR and hemodynamics was explored based on a simulated longitudinal experiment. Wall shear stress, flow patterns and vessel wall displacement were computed from these models. Pearson correlation analysis was performed to elucidate the relationship between the ASR and wall shear stress. The correlation of the ASR and flow velocity was also computed and analyzed.

**Results:**

The experiment results showed that there was a significant increase in IA area exposed to low WSS once the ASR > 0.7, and the flow became slower and the blood was more difficult to flow into the aneurysm as the ASR increased. Meanwhile, the results also indicated that average blood flow velocity and WSS had strongly negative correlations with the ASR (r = −0.938 and −0.925, respectively). A narrower impingement region and a more concentrated inflow jet appeared as the ASR increased, and the large local deformation at aneurysm apex could be found as the ASR >1.7 or 0.7 < the ASR <1.0.

**Conclusion:**

Hemodynamic characteristics varied with the ASR. Besides, it is helpful to further explore the relationship between morphologies and hemodynamics based on a longitudinal simulation by building a series of patient-specific aneurysm scaled models applying our proposed IA scaling algorithm.

## Background

Intracranial aneurysms (IAs) are pathological dilatations of cerebral vascular wall which are mostly located at the bifurcations of Circle of Willis [1-3]. Ruptured intracranial aneurysms usually lead to subarachnoid hemorrhage, resulting in considerable morbidity and mortality [4-7]. Therefore, it is very important and essential for clinicians to evaluate and predict intracranial aneurysm rupture risk, which is helpful in preoperative planning and preemptive treatments of IAs.

The morphological parameters (e.g. shape, size and location) have been proved to have impacts on the hemodynamic characteristics in IAs, which can be further used for the prediction of IA rupture risk [8]. The IA size has been widely considered as one of the key geometric parameters for the clinical assessment of IA rupture risk [9]. Besides, reports of International Study Group of Unruptured Intracranial Aneurysms (ISUIA) and other clinical reports indicated that the evaluation of IA rupture risk and the selection of cerebral surgical treatments generally depend on the IA size index with the evidence that the IA rupture risk increases as the IA enlarges [10-12]. Usually, IAs with a size of lager than 10 mm are regarded to be at a high rupture risk. On the other hand, some previous clinical studies showed that a lot of ruptured IAs had a size of smaller than 10 mm [13,14]. To date, there is still a controversy on the relationship between IA size and IA rupture risk, which need to be further investigated with more reliable methods and more concrete qualitative and quantitative analysis.

In order to explore the effect of IA size indices (such as aspect ratio defined as the IA height divided by the IA neck width) on hemodynamic parameters and the associated ability of predicting the IA rupture risk, many experiments based on computational fluid dynamics (CFD) have been carried out with real patient-based IA models and virtual IA models [15,16]. High average pressure was observed acting on the vessel wall within real patient IA models when the IA had a large aspect ratio [17]. The aspect ratio was also applied to identify the ruptured IA with a classification accuracy of nearly 66% based on an experiment including 55 real patient IAs (31 ruptured and 24 unruptured) [18]. However, no significant impact of IA aspect ratio on WSS was observed in 34 real unruptured and ruptured IA models [19]. The results of experiments based on real patient models were affected by the parent vessel geometry and IA shape and size, which led to the difficulty of investigating the impact of a single morphological parameter on hemodynamic characteristics. Meanwhile, the measurements of IA height and neck width in the computation of classical aspect ratio depend on the rotation of IA 3D images, so different values of aspect ratio may be obtained even for the same IA model [20]. Experiments with virtual expansion of IA could hold the parent vessel geometry invariant and was helpful for observing the impact of aspect ratio on rupture risk effectively. A study based on a series of simple virtual scaling spherical IA models, which consisted of lateral aneurysms on tubes with 3 mm diameter representing large arteries, was carried out. And the results showed that the value of WSS acting on dome area increased with the decreasing dome-neck ratio. In addition, by just changing the depth of an aneurysm while keeping the neck width constant, idealized IA models with different aspect ratios were built to explore the relationship between aspect ratio and hemodynamics [21]. Also, spherical and ‘egg-shaped’ patient-specific aneurysm models of varying aspect ratios were created to analyze the effects of aspect ratio on hemodynamics. The aneurysm sac was firstly separated from the real parent vessel and then integrated with an invariant idealized parent vessel. Lastly, a significant correlation between aspect ratio and energy loss was found [22].

In this paper, to better evaluate the relationship between the aneurysm size index and hemodynamic characteristics of IAs, we developed a size ratio scaling algorithm to create a series of aneurysm geometric models with different aneurysm size ratios (ASR) from a patient-specific IA model. Then, we applied a fully coupled fluid–structure interaction (FSI) simulation to obtain the WSS, flow patterns and wall displacement of the scaled IA models under pulsatile blood flow conditions. Analysis of hemodynamics among these scaled models is expected to give an insight into the relationship between ASR and intra-aneurysmal hemodynamics.

The paper is structured as follows: In Background section, previous studies and our work were briefly presented in the introduction section; Methods section described the details of the proposed scaling method, the construction of patient-specific models and the numerical simulation of IA models; Results was elucidated in Results section; Discussion was presented in the last section of the paper.

## Methods

### The original model

**Figure 1:**
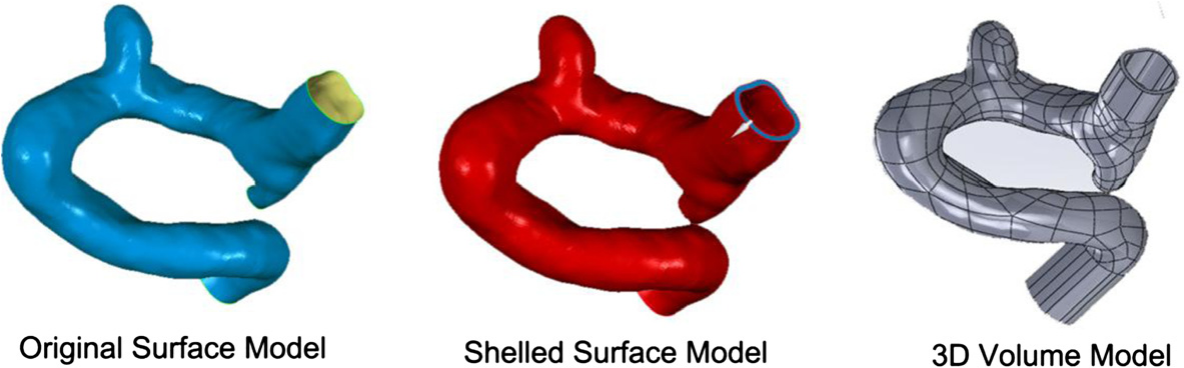
**The reconstruction of original aneurysm model.** The generations of original surface model, shelled surface model and 3D volume model are the main procedures in the reconstruction of aneurysm model based on a patient-specific aneurysm data.

Patient-specific 3DRA images were acquired on a GE LCV + Digital Subtraction system (LCV; GE Medical Systems) during a 200° rotation at a rate of 8.8 frames/s. The 88 projection images were converted into a 3D dataset using isotropic voxels on a dedicated GE workstation (Advantage Unix; GE Medical Systems). The raw DICOM file was firstly imported into the Mimics 10.0 software (Belgium Materialize company). Then the geometry of IA was extracted using an image cropping threshold, which was set manually, and further converted into a triangulated surface model. The blood vessel wall was constructed by extracting the surface along the normal direction of the wall in Geomagic 2012 software (Raindrop Geomagic, Durham, USA). The surface contour model was transformed into a 3D-soildvolume model by using the SolidWorks 2012 software (SolidWorks Corp, Concord, MA). The original model was thus constructed (Figure 1). The participants gave their informed consents and the Ethics Committee of Beijing Tiantan Hospital Affiliated to Capital Medical University approved the protocol of this study.

### The scaling models

**Figure 2:**
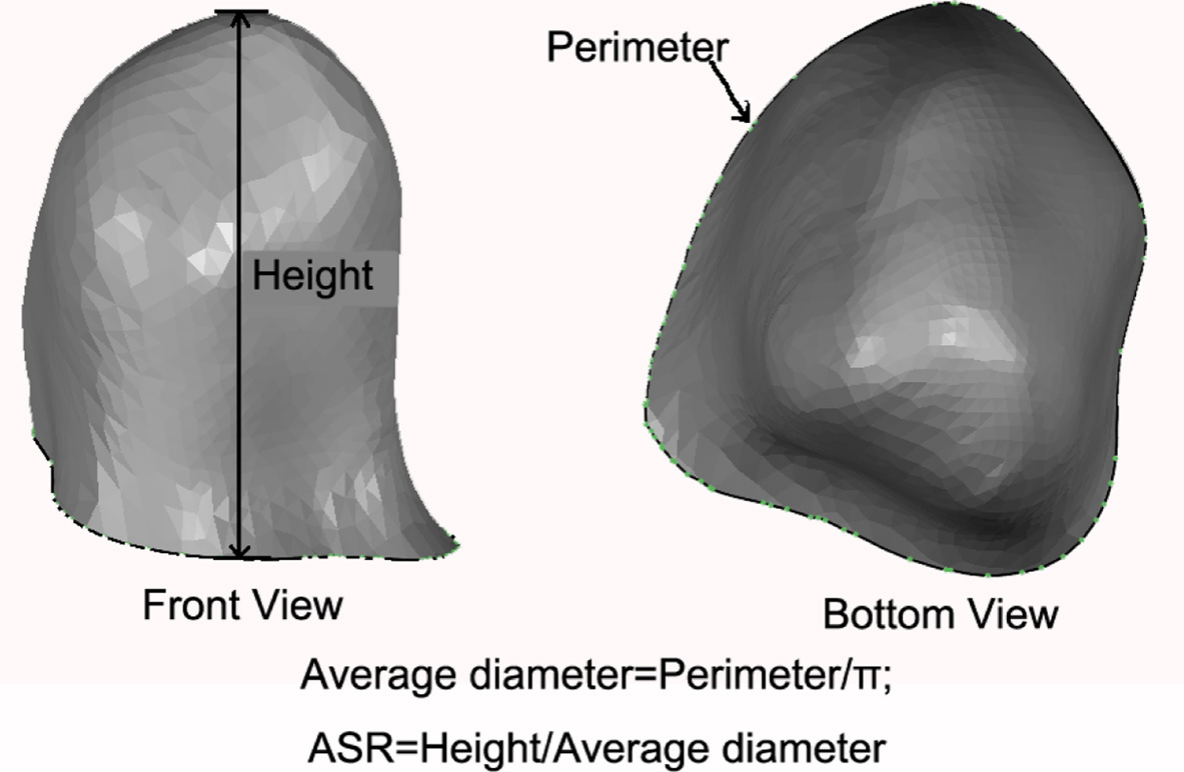
**The definition of ASR (Aneurysm Size Ratio).** The height is the perpendicular distance from the neck plane to the apex of the aneurysm sacculus, and the average diameter is computed from the perimeter of the aneurysm neck.

Firstly, we defined an IA size index termed ASR (Aneurysm Size Ratio, ASR), which was similar to the aspect ratio. The ASR was defined as the IA perpendicular height divided by the average neck diameter (Figure 2).

**Figure 3:**
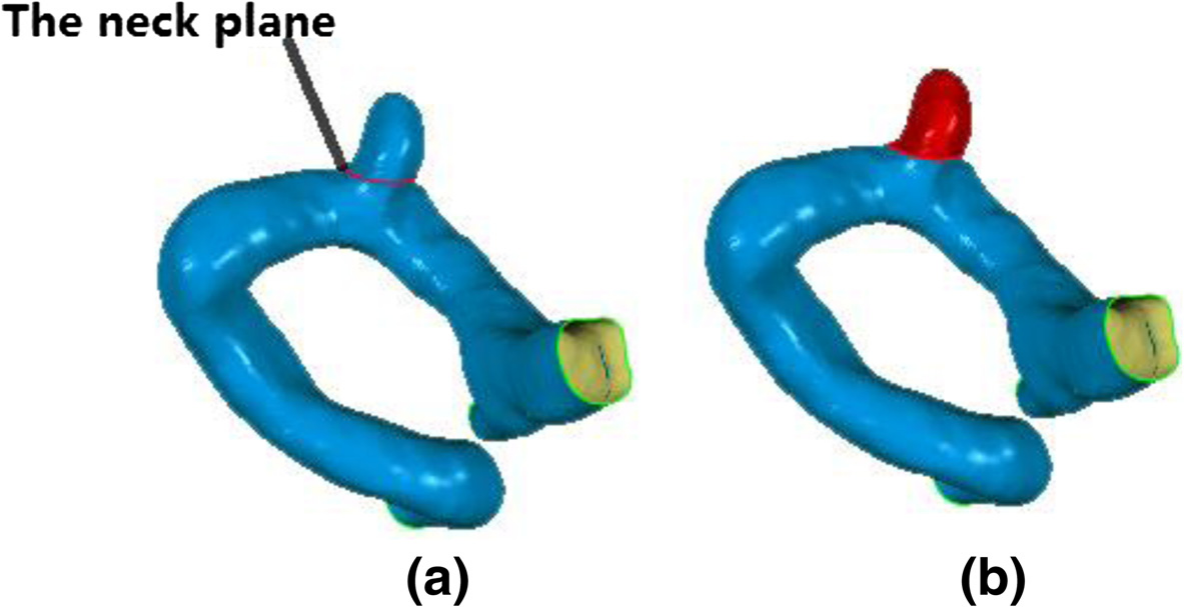
**The neck cutting plane and the aneurysm sac separated from the parent vessel. (a)** Cutting plane which was used to separate the aneurysm sac and parent vessel; **(b)** Separated aneurysm sac which would be scaled in the experiment (shown in red).

By scaling up or down the IA sacculus of the original patient-specific model, a series of scaled models with different ASRs were created, while other morphological parameters were kept constant. The details of the scaling method are presented as follows:

1. In order to change the size of the IA sac while keeping the parent vessel unchanged, we firstly determined the aneurysm neck cutting plane at which a clip was expected to be implanted for a surgical treatment of the patient. The cutting plane was used to separate aneurysm from the parent vessel. Figure 3 shows the neck cutting plane and the aneurysm sac isolated from the parent vessel respectively.

2. A new coordinate system (*XYZ*) was established through a rigid transformation of the original coordinates (*X*, *Y*, *Z*) in Geomagic 2012 software (Raindrop Geomagic, Durham, USA) such that the aneurysm neck plane was parallel to the XY plane in the new coordinates and *Z*-value of the aneurysm neck plane was defined as *d*. The rigid transformation applied is expressed below:

(1) $$\begin{array}{l} \left[\begin{array}{c} x\\ y\\ z\\ 1 \end{array}\right]=\left[\begin{array}{cccc} 1 & 0 & 0 & p\\ 0 & 1 & 0 & q\\ 0 & 0 & 1 & r\\ 0 & 0 & 0 & 1 \end{array}\right]\times \left[\begin{array}{cccc} \cos\text{Φ} & \sin\text{Φ} & 0 & 0\\ -\sin\text{Φ} & \cos\text{Φ} & 0 & 0\\ 0 & 0 & 1 & 0\\ 0 & 0 & 0 & 1 \end{array}\right]\times \left[\begin{array}{cccc} \cos\omega & 0 & -\sin\omega & 0\\ 0 & 1 & 0 & 0\\ \sin\omega & 0 & \cos\omega & 0\\ 0 & 0 & 0 & 1 \end{array}\right]\\ \;\times \left[\begin{array}{cccc} 1 & 0 & 0 & 0\\ 0 & \cos\theta & \sin\theta & 0\\ 0 & -\sin\theta & \cos\theta & 0\\ 0 & 0 & 0 & 1 \end{array}\right]\times \left[\begin{array}{c} x^{’}\\ y^{’}\\ z^{’}\\ 1 \end{array}\right] \end{array}$$

where *p*, *q*, *r* represente the displacements along *x*, *y*, *z* coordinates, respectively, and *Φ*, *ω*, *θ* represente the angles rotating around the *x*, *y*, *z* coordinates, respectively.

3. The geometric center of the aneurysms neck was obtained by using Geometric 12.0 software (Raindrop Geomagic, Durham, USA), and represented by (*x*_*m*_, *y*_*m*_, *z*_*m*_) (*z*_*m*_ = *d*).

4. In order to obtain the scaled models of the aneurysms sac, we linearly scaled the sac of the original aneurysms model by the following scaling transformation.

(2) $$\begin{array}{l} \left[\begin{array}{l} x_{t}\\ y_{t}\\ z_{t} \end{array}\right]=\left[\begin{array}{ccc} 1+\frac{a\times \left(z-z_{m}\right)\times \left(k-1\right)}{h} & 0 & 0\\ 0 & 1+\frac{b\times \left(z-z_{m}\right)\times \left(k-1\right)}{h} & 0\\ 0 & 0 & 1+\frac{c\times \left(z-z_{m}\right)\times \left(k-1\right)}{h} \end{array}\right]\\ \;\times \left[\begin{array}{l} x-x_{m}\\ y-y_{m}\\ z-z_{m} \end{array}\right];\left(z>z_{m}\right) \end{array}$$

where (*x*, *y*, *z*) is the pre-transformed point, and (*x*_*t*_, *y*_*t*_, *z*_*t*_) is the post-transformed point in the same coordinate system (*XYZ*). *h* is the perpendicular height of the aneurysms from the neck plane to the aneurysm dome. *k* is the scaling factor which represents the ratio of the height of the scaled IA sac model to that of the original sac.*a*, *b*, *c* are the scaling factors along *x*, *y*, *z* coordinates, respectively.

5. The scaled IA sac and parent vessels were saved as an integral data in the format of STL for later FSI analysis.

**Figure 4:**
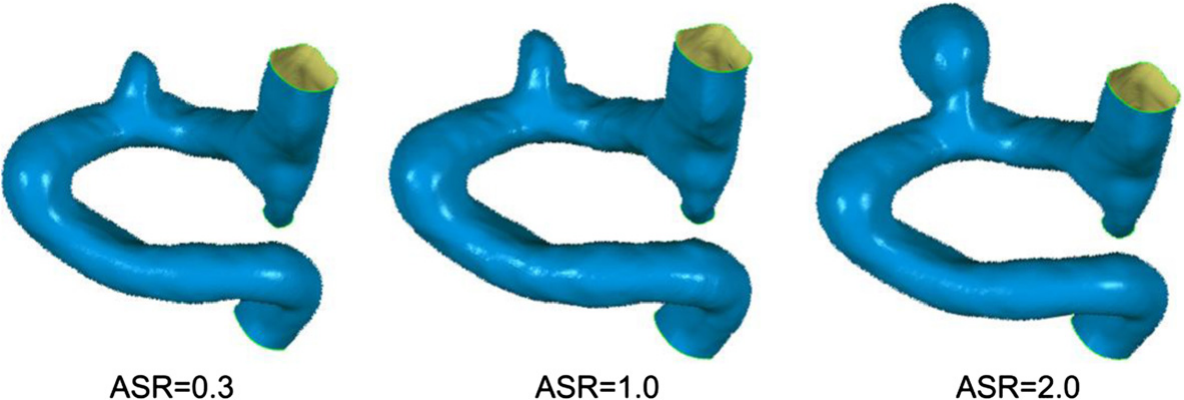
**The illustration of scaled models.** The scaling procedure is performed by changing the ASR of the aneurysm, three images are presented with ASR of 0.3, 1.0, and 2.0, respecitively.

In this work, the parameters *a*, *b*, *c* were set to be 2, 2, 1, respectively. The ASR was scaled up or down by adjusting *k* value, and the scaled models with ASRs of 0.3, 0.5, 0.7, 1.0, 1.3, 1.5, 1.7 and 2.0 were created. The choice of the ASR parameters in this experiment was based on the ASR distribution of patient datasets from Beijing Tiantan Hospital and suggestions of clinicians. The scaling mechanism was developed based on the phenomenon that expansion rate of aneurysm increased monotonically from the aneurysm sac neck to the fundus during the progression of aneurysm expansion [23]. In all the scaled models, the parent artery and the aneurysm neck were kept constant. Figure 4 shows the scaled models.

### Numerical modeling and boundary conditions

**Figure 5:**
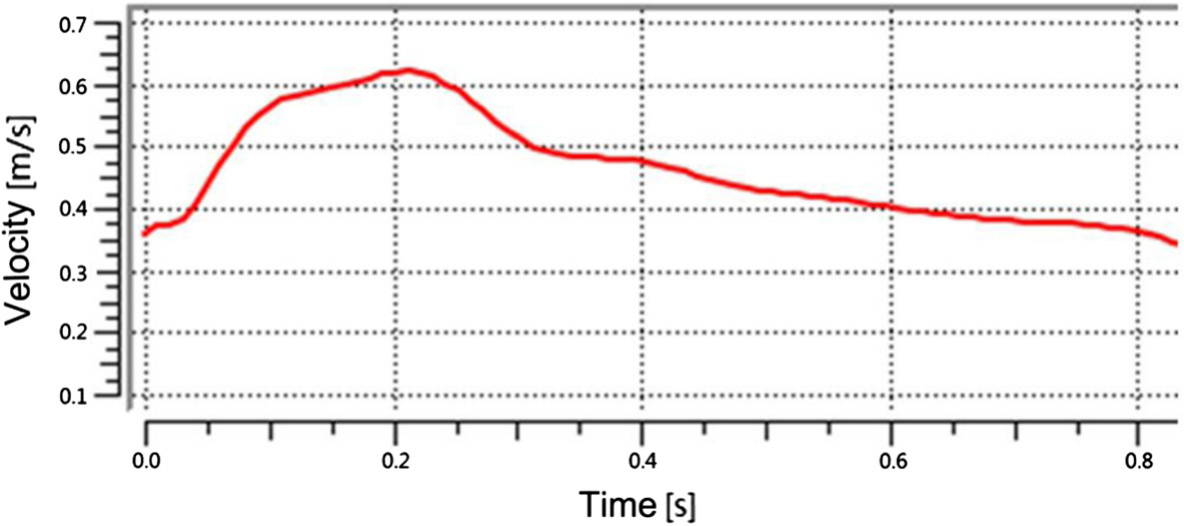
Physiologic flow conditions derived from TCD measurements on a normal subject.

There is no significant difference in the fluid fields of Newtonian and Non-Newtonian fluid models [24]. Aenis M et al. found that the error caused by the Non-Newtonian fluid model was less than 2% if the vessel diameter was larger than 0.5 mm [25]. In this experiment, Newtonian fluid was used in the IA numerical models due to the fact that the inlet diameter of the vessel was about 3.478 mm and the two outlet diameters were about 2.832 mm and 1.264 mm, respectively. The Reynolds number based on the entrance velocity of the inlet was 1095. If the average Reynolds number was smaller than 2300, the fluid was considered to be laminar flow. The blood was assumed to be a Newtonian fluid of uniform, isotropy, incompressible, unsteady, and laminar flow with a density of 1050 kg/m^3^ and a dynamic viscosity of 0.0035 Pa [26]. The blood flow varied throughout the cardiac cycle, so the inlet boundary condition was usually assumed to be pulsating flow. The flow velocity could be measured through the Transcranial Doppler (TCD) examination on a normal subject. Then, a flow velocity curve within a cardiac cycle (0.8 s) could be obtained through a nonlinear fitting method (as shown in Figure 5). The vessels were modeled to be a hyperelastic and isotropic material with Young’s modulus of 1.2 MPa, a Poisson’s ratio of 0.45 and a density of 1150 kg/m^3^[27]. We extended the outlet of the patient IA at the distal end in the normal downstream direction to 30 times the size of the vessel diameter, which was sufficient for the recovery of blood pressure at the IA. A zero pressure gradient in the flow direction was used at the outlets. No-slip flow boundary was imposed on the artery and aneurysm wall. For a longitudinal comparison, the same boundary condition was applied to all the scaled models (including the original model).

### Grid generation and computational tools

The mesh generation was implemented with ANSYS workbench 13.0 software (ANSYS Inc, Canonsburg, PA, USA). The fluid model was constructed with unstructured tetrahedral shell element and the solid model was created with hexahedral shell element. The fluid–solid interface mesh near the arterial wall consisted of prismatic shell elements. In addition, the number of elements was set to be 800,000 based on the mesh independency test, which showed that the changes in the hemodynamics parameters (e.g. WSS and flow velocity) were less than 5%. In this work, the number of elements in all the scaled IAs models ranged from 800,000 to 10,000,000.

The fluid–structure interaction simulation was carried out by using finite-volume software (ANSYS Workbench 13.0, ANSYS Inc, Canonsburg, PA, USA). The workstation for ANSYS software consisted of two Intel (R) Xeon (R) processors of 2.40GHz, a RAM of 24.0GB, and a Windows operating system (version 7). The computation time was approximately 4.6 CPU hours for each scaled IA model.

### Correlation analysis

**Figure 6:**
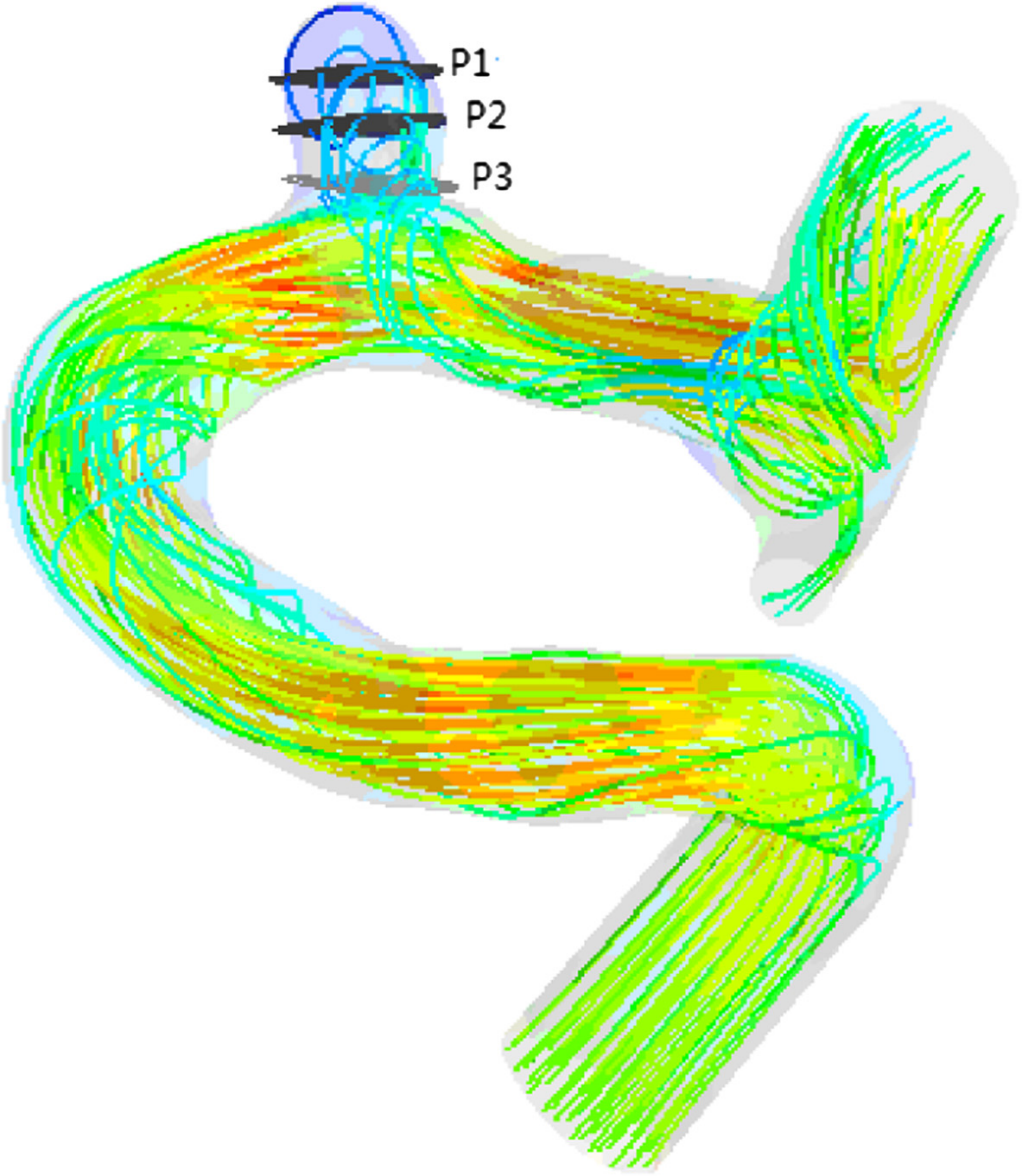
**Three planes in the aneurysm sac for the quantitative analyses.** Three planes in the aneurysm sac for the quantitative analyses were chosen at the neck (P3), the middle (P2), and the top (P1) of the aneurysm, which divided the aneurysm sac into three equal parts.

Blood flow patterns and WSS in each aneurysm models were obtained from three measurement planes which divided the aneurysm sac into three parts at the level of the aneurysm neck plane, middle plane and top plane, respectively (Figure 6). The average flow velocity and average WSS of the three measurement planes were also calculated for the analysis of correlations with the ASR by using the SPSS 16.0 software (SPSS Inc Chicago, Illinois).

## Results

The numerical simulations for all IA models were carried out under the same boundary conditions. Fully coupled fluid–structure interaction (FSI) simulation was applied to obtain the wall shear stress, flow patterns and wall displacement.

### Wall shear stress

**Figure 7:**
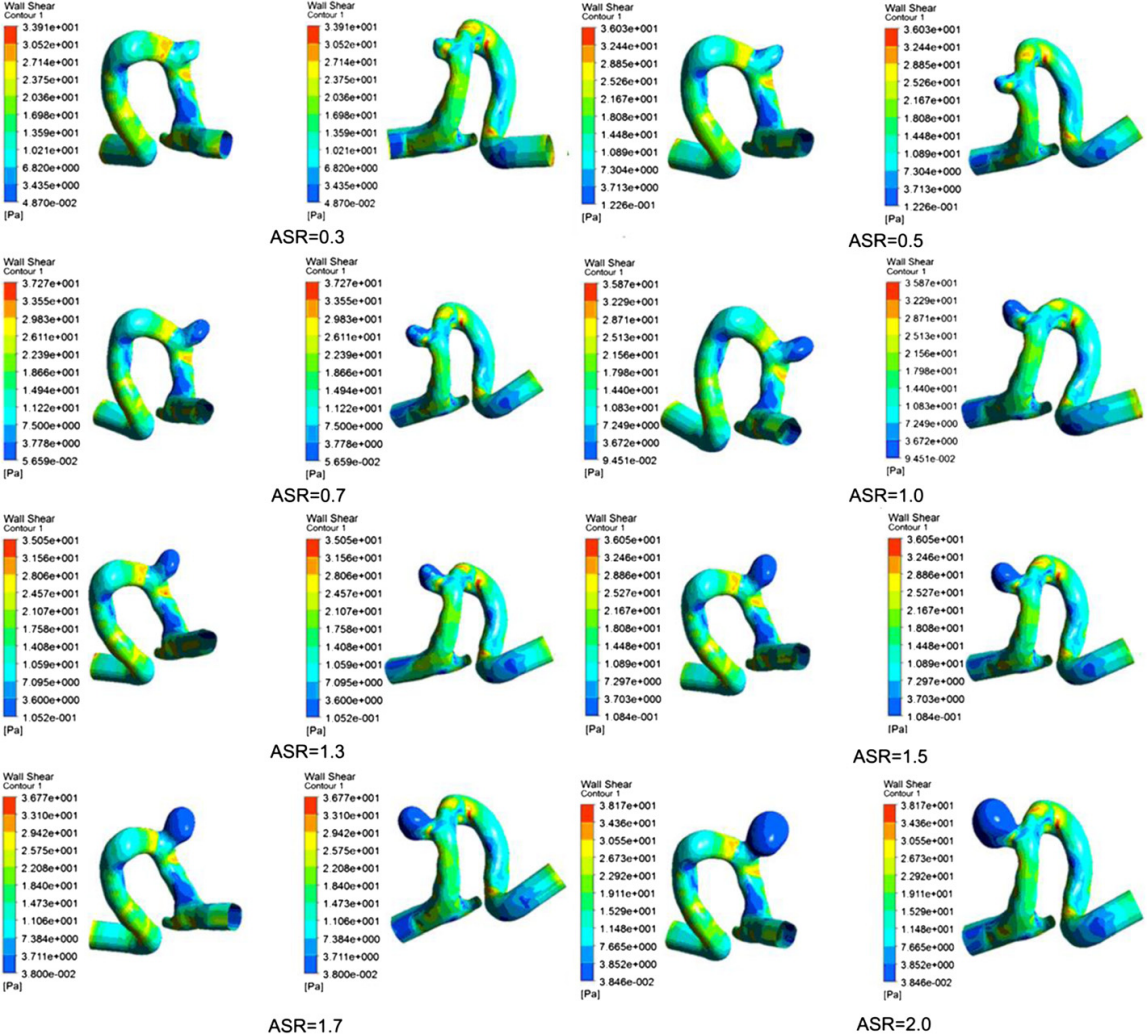
**The distribution of WSS at peak systole.** The image described the location of the aneurysm sac. And the remaining images showed WSS distribution of the scaled models including the original aneurysm. The WSS distributions showed that there were significant differences between different aneurysm models.

**Figure 8:**
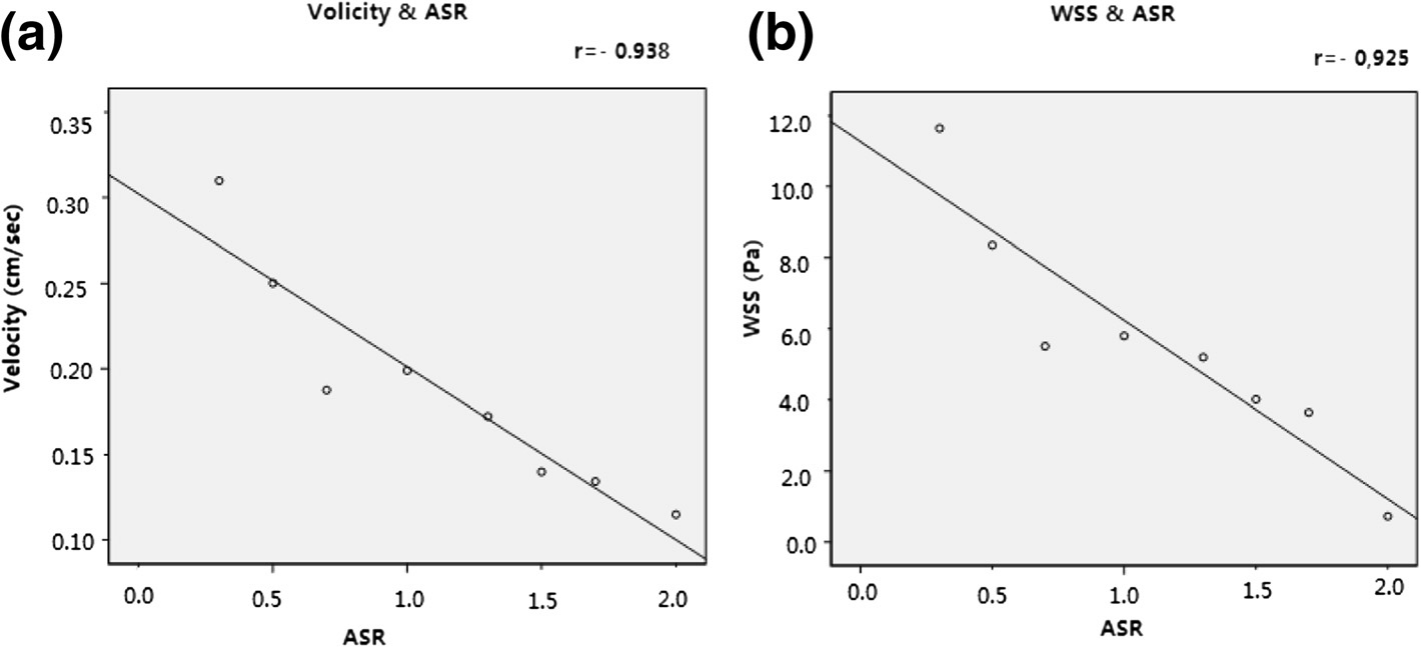
**Correlation between average flow velocity and ASR**; **correlation between average WSS and ASR.** Significant negative correlations were observed with **(a)** r = −0.938 and **(b)** r = −0.925, respectively.

We obtained the distribution of WSS acting on the aneurysm vessel wall at systole (see Figure 7). The distribution of WSS was uneven for each IA model with a specific ASR (including the original model), while the maximum WSS (up to 30 Pa) was observed at the distal of the aneurysm neck for all the models. The distribution area of low WSS (<0.4 Pa) in the aneurysm sac expanded and the value of low WSS decreased as the ASR increased. Besides, as shown in Figure 8a, a significant negative correlation (r = −0.925) was found between the ASR and the average WSS computed from three predefined measurement planes within the aneurysm sac.

### Blood flows

**Figure 9:**
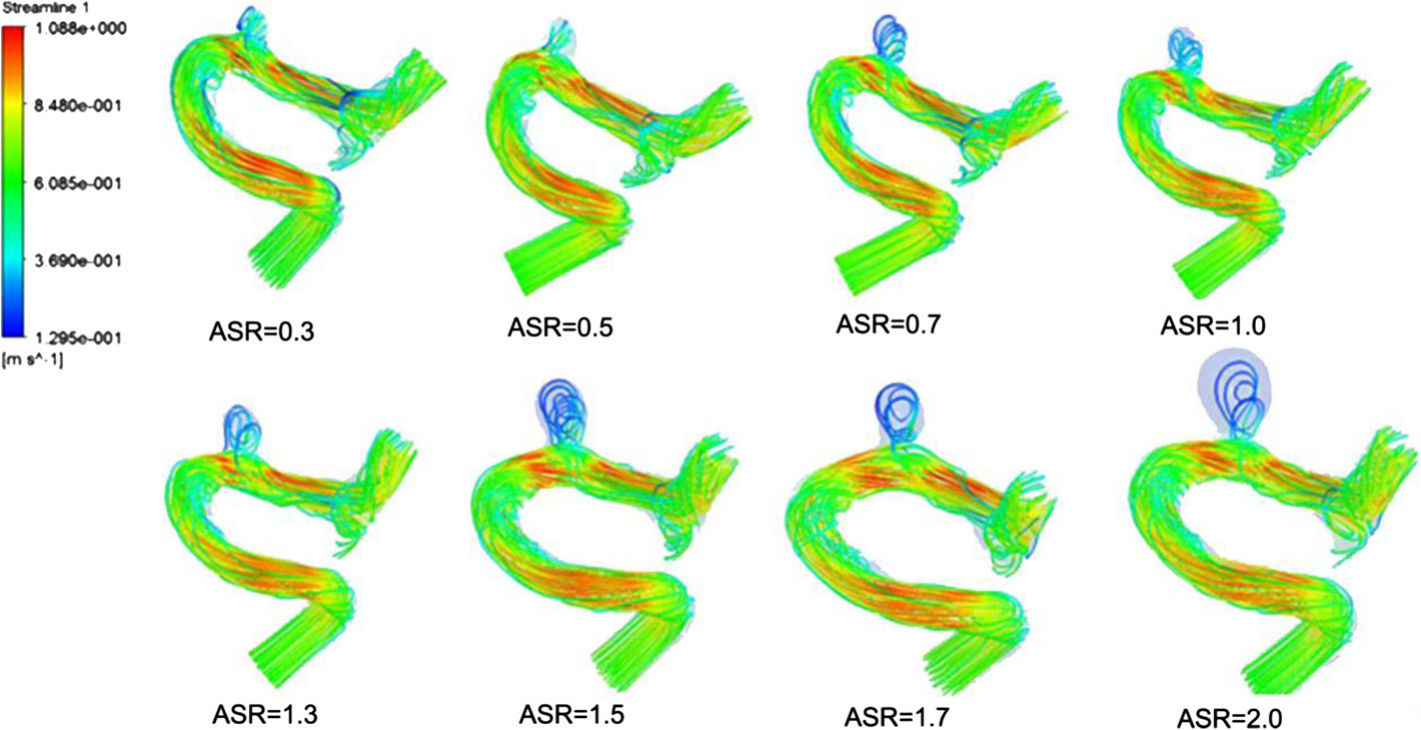
**Blood flow streamlines within the aneurysm models at peak systole.** The image described the flow velocity, quantity, and vortex in all the scaled models including the original model. Obvious variations could be observed with the changes of ASR.

**Figure 10:**
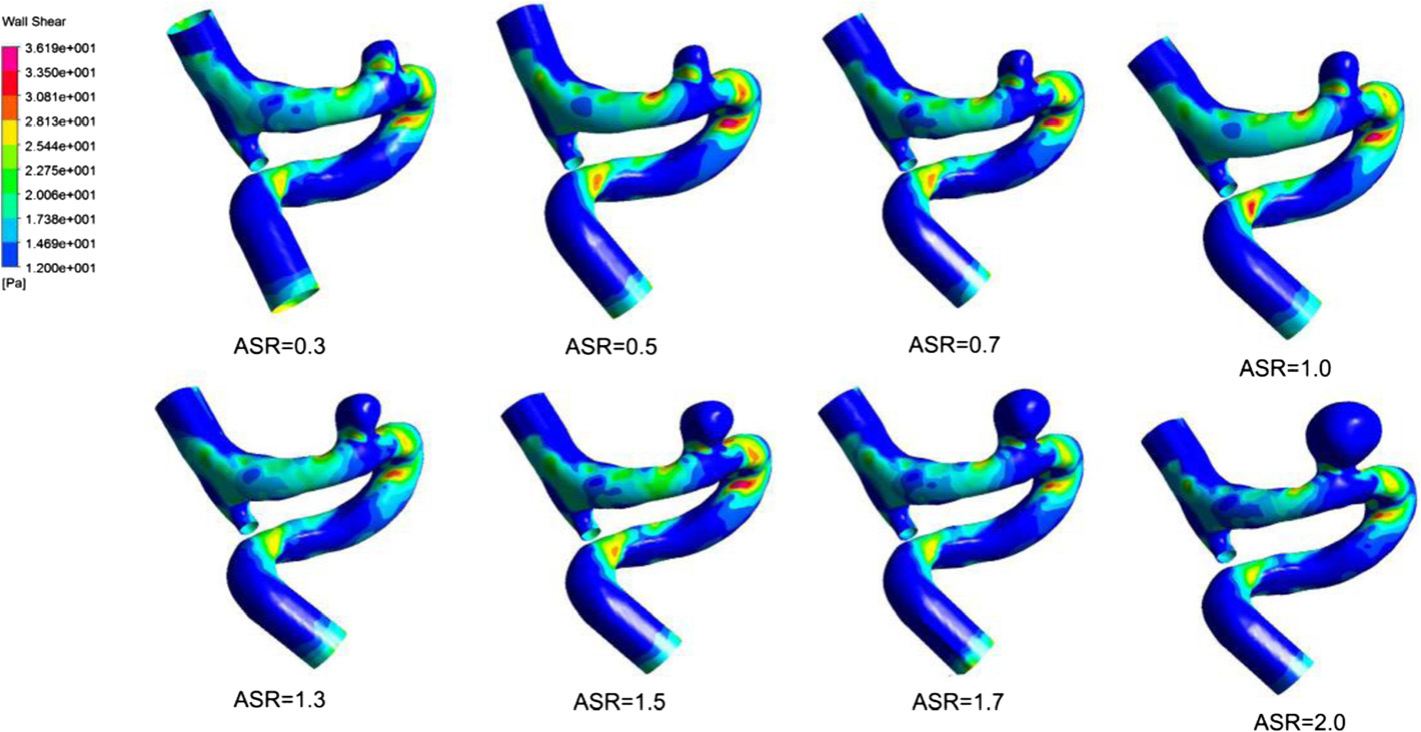
**The changes of impingement zone of aneurysm models with different ASRs.** The impingement became narrower as the ASR increased especially when the ASR > 1.5.

**Figure 11:**
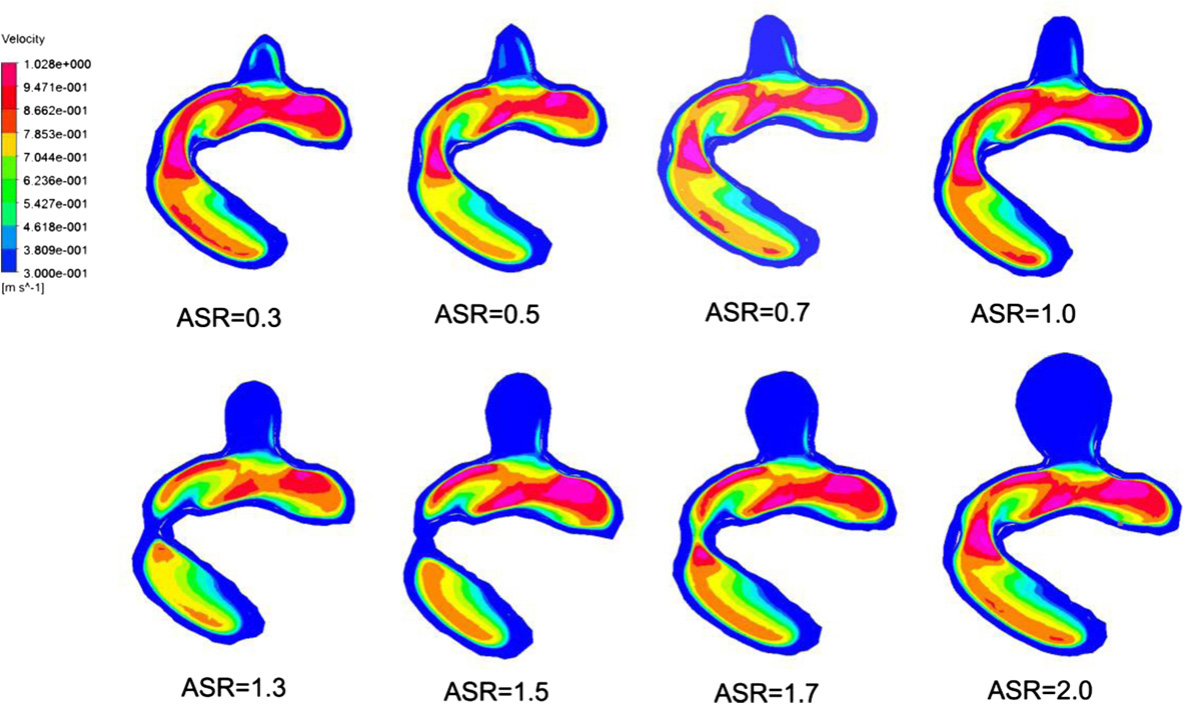
**The changes of inflow jet of aneurysm models with different ASRs.** The cutting plane for illustration of inflow jets was set by manually choosing three points on the original parent vessel surface and was applied for all the scaled models. The inflow jet became concentrated as the ASR increased especially when the ASR > 1.5.

We found that a part of blood flowed into the aneurysm sac while the remaining blood kept flowing along the parent vessel (as shown in Figure 9). Meanwhile, significant differences in blood flow velocity and vortex were observed in IA models with different ASRs. As the ASR increased, the blood flow patterns became more complex. In the aneurysm sac, with the increasing ASR, the vortices appeared gradually, the flow velocity decreased, and the blood flow into the aneurysm sac became smaller, especially when the ASR was larger than 1.5. The ASR and the average flow velocity extracted from the three measurement planes were found to have a strongly negative correlation. Beside, as shown in Figures 10 and 11, the larger the ASR, the narrower the impingement zone and the more concentrated the inflow jet. The inflow jet consisted of parallel inflow with high speed compared with other parts in the aneurysm. The impingement zone was the region on the aneurysm wall where the inflow jet was seen to impact the wall for the first time with high speed [28]. In addition, the results showed that the intra-aneurysmal hemodynamic characteristics were greatly affected by the ASR, and the flow velocity showed a strongly negative correlation (r = − 0.938) with the ASR (Figure 8b).

### Wall displacement

**Figure 12:**
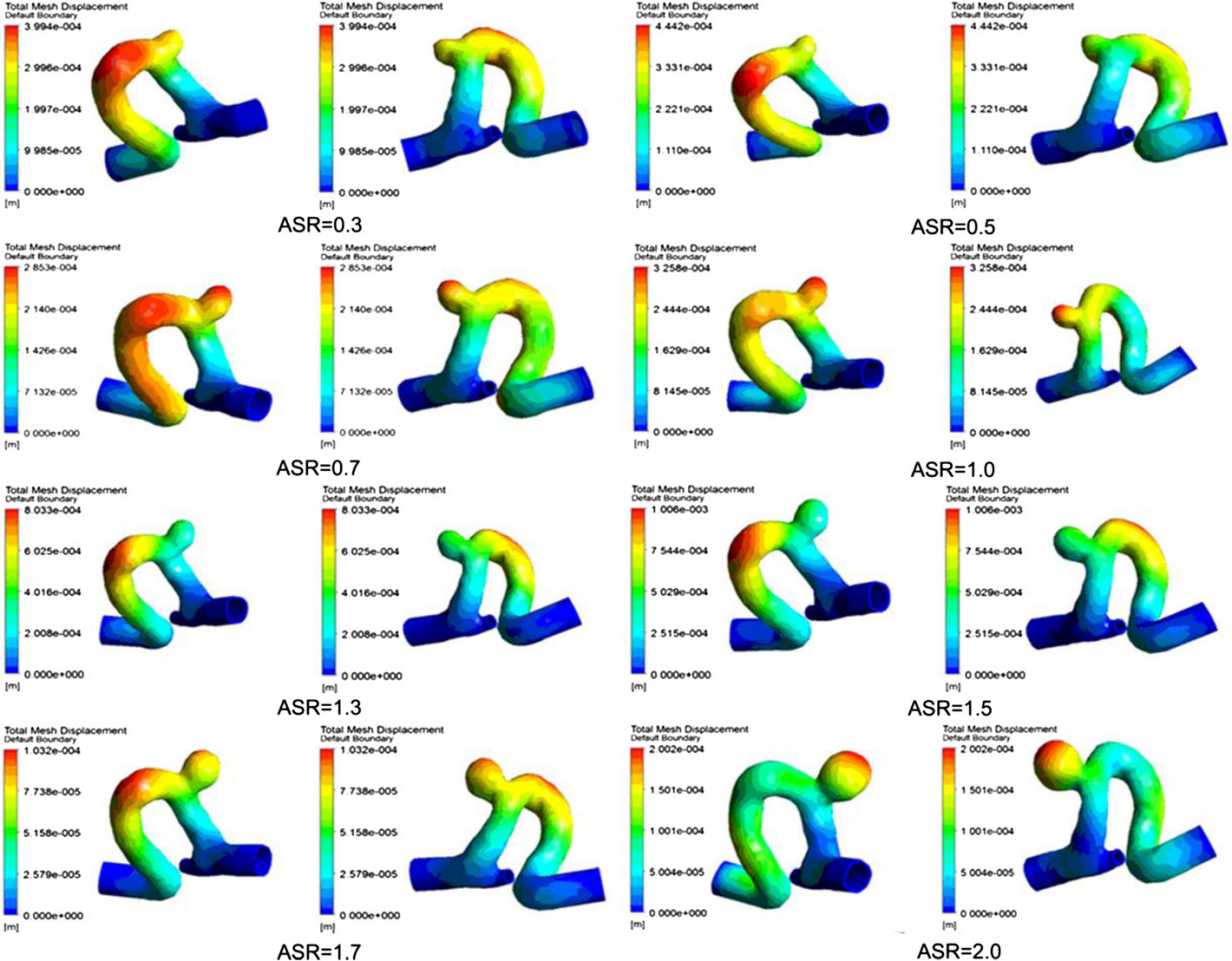
**The wall displacements of the vessel within all the aneurysm models.** The large displacements occur at the apexes of the scaled aneurysm models which are shown obviously especially when ASR = 0.7, 1.0, 1.5.

Figure 12 shows the wall displacement at systole. In this experiment, the value of wall displacement was approximately two folds of the vessel wall thickness (0.3 mm setted in all the numerical models), and the maximum wall displacement observed was about 0.5 mm. It was noted that the location of maximum wall displacement in IA models with different ASRs varied significantly. When the ASR > 1.7 or 0.7 < the ASR < 1.0, the maximum wall displacement occurred at the apex of the aneurysm with a deformation range of 0.2-0.4 mm.

## Discussion

In current studies, longitudinal experiments have been adopted to explore the relationship between the aspect ratio and hemodynamics based on real IA growth models or virtual IA scaled models [16]. However, there were few tracking time points of patients with IAs for longitudinal experiments due to the fact that a timely clinical treatment should be carried out upon the diagnosis of IAs. Besides, individual differences of IAs can lead to different hemodynamic changes which might result in different conclusions especially in the longitudinal experiments with small sample size. As for virtual IA models, previous works mostly focused on a series of idealized IA models [22]. In this paper, we proposed a novel scaling method of IAs based on patient-specific 3DRA data to investigate the impact of ASR on some hemodynamic parameters, which could be further used to predict the IA rupture risk. The ASR could avoid the uncertainty of classical aspect ratio due to the rotation of aneurysm image. Besides, the expansion of IAs was a linear scaling of the patient-specific models by changing the ASR and keeping other morphological parameters constant, which might contribute to the longitudinal simulated experiments of IAs.

In last decades, a close relationship between hemodynamic parameters and the rupture risk of IAs has been found. WSS was an important hemodynamic parameter related to the remodeling of vessel wall. A negative effect of low WSS on vascular endothelial cell has been reported, suggesting that the low WSS might be a key factor related to the growth and rupture of IAs [29]. A low WSS was found at the regions where an aneurysm usually was located and the rupture risk increased with the growth of the aneurysm [30-32]. Besides, a vitro experiment demonstrated that the degeneration of endothelial cell occurred due to the exposure of vessel endothelium and the low WSS (< 0.4 Pa) [31,33,34]. The results of our experiments showed that the area of low WSS (< 0.4 Pa) became larger as the ASR of IAs increased, which implied a negative correlation between the WSS and the ASR. In addition, previous works showed that large deformation of the vessel wall within the aneurysm was located at the dome of IA [35]. In our experiment, when the ASR > 1.7 or 0.7 < the ASR < 1.0, the maximum deformation appeared at the apex of the aneurysm sac with a range of 0.2-0.4 mm. Based on the clinical experience that large deformation of the vessel wall increases rupture risk [35], we concluded that the rupture risk was high when the ASR was in a particular range. A comparison between a numerical simulation analysis and the clinical disease history showed that un-ruptured IAs commonly had a simple and stable flow pattern with a large impingement zone and large inflow jet region. On the other hand, a disturbed flow pattern with a small impingement zone and small inflow jet region were usually found in ruptured IAs [36]. Complex flow patterns, which were considered to increase the rupture risk of IAs [30,37], were observed as the ASR became larger in our experiments. Based on the fact that slow blood velocity could cause the low WSS acting on vessel wall, which led to the final rupture of IAs, we speculated that the ASR, which related to the flow patterns and WSS, could be regarded as an IA rupture risk factor.

In this work, a unified blood viscosity was applied to all the scaled IA models (including the original model), which was not so accurate as the real blood viscosity is affected by the haematocrit, osmotic pressure and temperature in a particular patient. As for the boundary conditions, the inlet flow velocity was set based on experiment records of a normal person through Doppler ultrasound. Due to the unchanged geometry of parent vessel, the same material properties and boundary conditions were used in all the scaled IA models for the investigation of hemodynamic variations caused by the changed ASR alone. The longitudinal analysis was focused on the differences of hemodynamics parameters spatial distribution other than the absolute values of hemodynamics, which was meaningful to some extent for IAs rupture risk stratification.

Effects of morphological and hemodynamic parameters of IAs on the rupture should be further studied based on numerous real patient-specific datasets, more reliable modelling techniques, more reliable numerical simulation procedures and more adequate qualitative and quantitative analysis. A further understanding of physiological mechanism can help us improve the expansion or scaling method, and the parameters in our proposed scaling method should be set based on more real patient longitudinal data. This work is an exploratory attempt of advanced virtual simulations of IAs in that a series of scaled IAs were constructed to investigate the relationship between the ASR and some hemodynamic parameters. We will continue this study to develop more sophisticated scaling methods of IAs to investigate the impacts of other shape indices (e.g. undulation, non-sphericity and ellipticity) on the hemodynamics and the rupture risk of IAs.

## Conclusion

Hemodynamic variations of a series of IA models with different ASRs showed that the ASR affected the hemodynamic characteristics including wall shear stress, flow patterns and vessel wall displacement. And since these hemodynamic parameters have impacts on the IA rupture risk, ASR can be regarded as an IA rupture risk factor. Besides, the scaling method proposed in this paper may provide the foundation for constructing more sophisticated expansion models of IAs, which will be helpful for the investigation of effects of aneurysm morphological parameters on hemodynamic characteristics.

## Abbreviations

ASR: Aneurysm Size Ratio; 3DRA: 3-D Rotational Angiography; IA: Intracranial Aneurysm; FSI: Fluid–structure Interaction; WSS: Wall Shear Stress.

## Competing interests

The author’s declare that they have no competing interests.

## Authors’ contributions

YL: made substantial contributions to the conception, design, analysis and interpretation of data, and has drafted the manuscript. HY and ZZ: made contributions to the design of the work and revision of the manuscript. YZ, YW and XY: made contributions to acquisition of data. HL: the corresponding author, made contributions to the conception and interpretation of data, have given the final approval of the version to be published. All authors read and approved the final manuscript.

## Authors’ information

The first author. There is only one first author named Yunling Long.
